# Rapid Enumeration of Active *Legionella pneumophila* in Freshwater Environments by the Microcolony Method Combined with Direct Fluorescent Antibody Staining

**DOI:** 10.1264/jsme2.ME11324

**Published:** 2012-03-23

**Authors:** Takashi Baba, Naoko Inoue, Nobuyasu Yamaguchi, Masao Nasu

**Affiliations:** 1 Graduate School of Pharmaceutical Sciences, Osaka University 1–6 Yamadaoka, Suita, Osaka 565–0871, Japan

**Keywords:** *Legionella pneumophila*, rapid enumeration, microcolony, growth activity, freshwater

## Abstract

In this study, a microcolony technique was combined with direct fluorescent antibody staining for the specific detection and enumeration of *Legionella pneumophila* in freshwater samples with growth activity. This method allowed the detection of active *L. pneumophila* (within 48 h) in 91 bath water samples collected from 30 bathing facilities, with similar sensitivity of a conventional plate-counting method. These results suggest that the microcolony method combined with fluorescent antibody staining could be useful as a monitoring technique for the prevention of Legionnaires’ disease through the early detection of *L. pneumophila* in freshwater.

Members of the genus *Legionella* are ubiquitous in freshwater environments and the vast majority of Legionnaires’ disease cases are due to *L. pneumophila*(5). Outbreaks of Legionnaires’ disease have been associated with a wide variety of freshwater sources, including cooling towers, fountains, hot tubs, spas and so on (3, 9, 15). In Japan, the reported incidences of Legionellosis have been steadily increasing and the main source of contamination is considered to be water from bathing facilities and spas (8, 10, 11).

The rapid monitoring of *Legionella* species in environmental water is essential for the prevention of Legionnaires’ disease outbreaks. When an increase of *Legionella* species is detected in environmental water, such as a water circulation system, rapid disinfection of the water leads to efficient control of Legionellosis outbreaks. The detection and enumeration of *Legionella* species have been typically performed using conventional culture methods; however, this approach is time-consuming, as an incubation period of up to 10 days is required for determining the number of *Legionella* spp.

In recent years, PCR-based methods have been used for the detection and quantification of *Legionella* spp. For example, a quantitative real-time PCR technique targeting the 16S rRNA gene and *mip* (macrophage infectivity potentiator) genes of *L. pneumophila* has been used to detect targeted cells in water samples (12). On the other hands, detection methods discriminating between live and dead cells are of major interest because viable *L. pneumophila* with growth activity are able to increase by the proliferation and cause infection outbreaks. Therefore, the development of a rapid enumeration method for the quantification of active *L. pneumophila* with growth activity is required for efficient water hygiene management and control of Legionellosis.

One such approach is the microcolony method, which is based on microscopic observation of the early stages of colony formation on selective culture medium and allows the enumeration of viable target cells by growth activity (6). Several studies have reported that most bacteria in the natural environment grow to the microcolony stage, while they hardly form visible colonies (1, 7, 14). In this study, we combined the microcolony method with fluorescent antibody staining for the identification and enumeration of active *L. pneumophila* in environmental water samples collected from 30 bathing facilities and spas (91 samples).

*L. pneumophila* (JCM7571) was used to optimize the microcolony-fluorescent antibody staining (MC-FA) method. *L. pneumophila* was grown on buffered charcoal yeast extract agar supplemented with α-ketoglutarate (BCYEα) agar media (Eiken Chemical Co. Ltd., Tokyo, Japan) and suspended in PBS. Approximately 10^6^ cells of *L. pneumophila* were trapped by vacuum on membrane filters (pore size: 0.2 μm, ANODISC 25; Whatman International, Ltd., Kent, UK), which were then transferred onto BCYEα agar medium and incubated at 37°C for 48 h. These filters were placed on filter paper (No. 2; Whatman International Ltd.) soaked with 4% formaldehyde to fix microcolonies at room temperature for 30 min. Membrane filters were then transferred onto filter paper saturated with sterile water, allowed to stand for 10 min, and then air-dried. For the enumeration of *L. pneumophila* microcolonies, membrane filters were stained with fluorescein isothiocyanate (FITC)-labeled anti-*L. pneumophila* antibodies (Monoclonal Technologies Inc., Alpharetta, GA, USA) as described below. The specificity of this fluorescent antibody was reported (4). Membrane filters were treated with 10 μl fluorescent antibodies diluted with 3% bovine serum albumin (BSA; Wako Pure Chemical Industries, Osaka, Japan) in PBS at 30°C for 30 min. Membrane filters were placed on filter paper soaked with PBS to rinse for 10 min and allowed to air dry. Microcolonies of *L. pneumophila* were counted by epifluorescent microscopy (E-400; Nikon, Tokyo, Japan) with 200×magnification.

Microcolony formation of *L. pneumophila* was monitored for a 48-h period (Fig. 1). After 32 h, the formed microcolonies were approximately 20 μm in diameter and reached approximately 100 μm in diameter after 48 h. The number of active *L. pneumophila* by the MC-FA method was (1.4±0.9)×10^6^ microcolony-forming units (mCFU) (100 mL)^−1^, while it was (1.8±0.7)×10^6^ CFU (100 mL)^−1^ by the conventional plate-counting method. There was no significant difference in the results obtained by the two methods. Fluorescent antibody staining allowed the simple and specific detection of *L. pneumophila* microcolonies. With the MC-FA method, only 48 h or less was required for the detection of microcolony formation of *L. pneumophila*, which is markedly shorter than the ten days required for the conventional plate-counting method. An additional advantage of this method is the counting accuracy; the target bacteria can be easily distinguished from contaminants (non-biological particles) included in environmental samples, because the formed microcolonies are markedly larger (>20 μm) than single cells or contaminants in the microscopic field.

After optimization of the MC-FA method, bath water samples were collected from 30 bathing facilities and spas in Japan from July 2004 to November 2006. Ninety-one samples were collected in sterilized plastic bottles and used immediately after sampling. In environmental samples, detection of *Legionella* cells in bath water samples by the conventional plate-counting method was performed according to JIS K 0350-50-10:2006. Samples of water (500 mL) were concentrated by filtration through a poly-carbonate filter (pore size: 0.2μm; Toyo-Roshi, Tokyo, Japan). After filtration, bacteria collected on membrane filters were resuspended in 5 mL sterilized water and sonicated for 1 min. The suspensions were heat treated at 50°C for 30 min to suppress the growth of non-*Legionella* bacteria and then serially diluted in sterile water. Subsequently, 100 μl of each sample was spread on Wadowsky-Yee-Okuda glycine-vancomycin-polymyxin B-amphotericin B supplemented with α-ketoglutarate (WYOα) medium (Nikken Bio Medical Laboratory, Kyoto, Japan) and incubated at 37°C for 7 days. *Legionella*-like colonies, which were grayish-white in color, were subcultured on both sheep blood and BCYEα agar media (Nikken Bio Medical Laboratory) and incubated at 37°C for 2 days for further verification. Colonies that grew on only BCYEα medium were subjected to slide agglutination tests using *Legionella* antisera kit (Denka Seiken, Tokyo, Japan) to determine the *Legionella* serogroup.

The number of *L. pneumophila* in each sample was also determined by the MC-FA method. Filtering, concentration, and heat treatment were performed by identical procedures to those described for the plate-counting method. Then, 1 mL of each sample was filtrated through a membrane filter and the filters were transferred onto WYOα medium and incubated at 37°C for 48 h. Fixation and staining were performed by identical procedures to those described previously. The whole area of each filter was scanned under an epifluorescent microscope with 200× magnification, and the lower detection limit was 1 mCFU (100 mL)^−1^.

In 91 bath water samples collected from bathing facilities and spas, 25 samples (28%) were positive by the MC-FA method, and 16 (17%) were positive by the plate-counting method. The number of colony-forming and microcolony-forming units in the positive samples, as determined by the plate-counting and the MC-FA method, respectively, are shown in Table 1. Nine samples were positive by the MC-FA method but negative by the plate-counting method, whereas the opposite was never found. In most bathing facilities, bath water was treated by detergents and chlorination, and *L. pneumophila* cells may have been damaged by treatment and impaired in their ability to form visible colonies on the selective medium. Furthermore, in environmental microbiology, it is recognized that many human pathogenic bacteria do not form visible colonies on conventional culture media when in the natural environments (2, 16).

On the other hand, in all positive samples by both methods (16 samples), a positive relationship was observed (r=0.791, *p*<0.01); in particular, for five samples containing a high concentration of active *L. pneumophila* (>100 CFU [100 mL]^−1^), the bacterial number by the MC-FA and plate-counting methods was close. We concluded that the MC-FA method is capable of rapidly identifying active *L. pneumophila* in water samples. These findings suggest that this MC-FA method could be useful as a rapid monitoring technique of *L. pneumophila* for the prevention of Legionnaires’ disease outbreaks.

It is expected that the MC-FA method described here can be applied for the detection of any target bacterium for which selective media and antibodies have been developed. Furthermore, this method with the Microcolony-Auto-Counting-System (13, 14) enables the routine bacterial monitoring of environmental samples.

## Figures and Tables

**Fig. 1:**
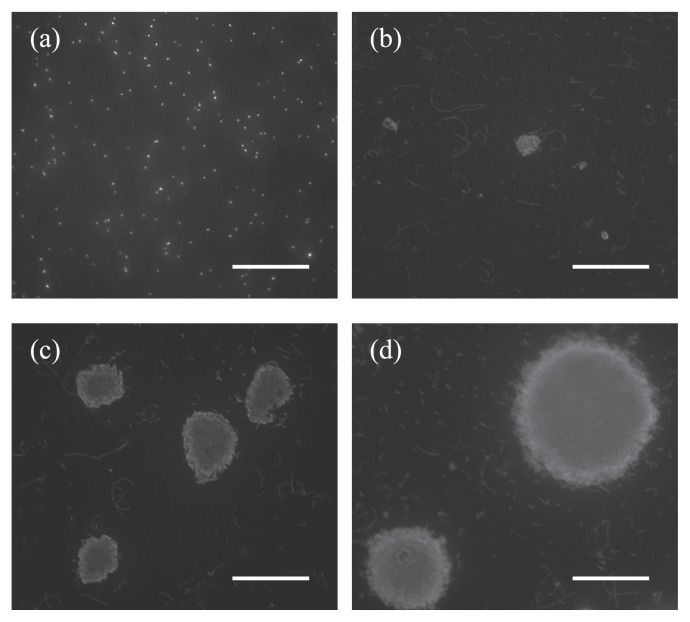
Epifluorescent micrographs of *Legionella pneumophila* microcolonies by the MC-FA method. *L. pneumophila* serogroup 1 (JCM7571) cells on membrane filters were cultured on BCYEα medium for the indicated time periods and subjected to staining with FITC-labeled anti-*L. pneumophila* antibodies. Before incubation (a), incubated for 24 hours (b), 32 hours (c), 48 hours (d). Scale bars are 50 μm.

**Table 1:** Quantification of *L. pneumophila* in bath water samples by the plate-counting and the microcolony-fluorescent antibody staining (MC-FA) method

Sample No.	Temperature (°C)	Cl (mg L^−1^)	Plate-counting method (CFU 100 mL^−1^)	MC-FA method^a^ (mCFU 100 mL^−1^)
11	38.0	<0.1	ND^b^	2
80	42.0	<0.1	ND	2
91	42.0	<0.1	ND	3
86	40.0	<0.1	ND	5
12	37.0	<0.1	ND	11
35	40.3	<0.1	ND	12
70	42.0	1.0	ND	13
64	40.0	<0.1	ND	18
36	41.3	<0.1	ND	114
22	38.0	<0.1	10	2
66	42.0	<0.1	10	3
31	41.4	<0.1	10	66
45	39.8	<0.1	20	7
44	40.7	<0.1	20	18
30	41.3	<0.1	20	57
46	40.9	<0.1	40	3
43	42.0	<0.1	40	12
29	40.2	<0.1	40	49
28	39.9	<0.1	50	41
32	42.5	<0.1	70	39
38	40.2	<0.1	180	171
34	40.0	<0.1	230	140
37	40.4	<0.1	670	980
68	42.0	<0.1	820	1010
39	28.6	<0.1	1200	1869

a Means of two replicates.

b Under detection limit (10 CFU 100 mL^−1^)

## References

[1] Baba T, Matsumoto R, Yamaguchi N, Nasu M (2009). Bacterial population dynamics in a reverse-osmosis water purification system determined by fluorescent staining and PCR-denaturing gradient gel electrophoresis. Microbes Environ.

[2] Bej A, Mahbubani MH, Atlas RM (1991). Detection of viable *Legionella pneumophila* in water by polymerase chain reaction and gene probe methods. Appl Environ Microbiol.

[3] Borella P, Montagna T, Stampi S (2005). *Legionella* contamination in hot water of Italian hotels. Appl Environ Microbiol.

[4] Delgado-Viscogliosi P, Simonart T, Parent V (2005). Rapid method for enumeration of viable *Legionella pneumophila* and other *Legionella* spp. in water. Appl Envion Microbiol.

[5] Fields BS, Benson RF, Besser RE (2002). *Legionella* and Legionnaires’ disease: 25 years of investigation. Clin Microbiol Rev.

[6] Kawai M, Yamaguchi N, Nasu M (1999). Rapid enumeration of physiologically active bacteria in purified water used in the pharmaceutical manufacturing process. J Appl Microbiol.

[7] Kenzaka T, Yamaguchi N, Utrarachkij F, Suthienkul O, Nasu M (2006). Rapid identification and enumeration of antibiotic resistant bacteria in urban canals by microcolony-fluorescence in situ hybridization. J Health Sci.

[8] Kura F, Amemura-Maegawa J, Yagita K, Endo T, Ikeno M, Tsuji H, Taguchi M, Kobayashi K, Ishii E, Watanabe H (2006). Outbreak of Legionnaires’ disease on cruise ship linked to spa-bath filter stones contaminated with *Legionella pneumophila* serogroup 5. Epidemiol Infect.

[9] Leoni E, De Luca G, Legnani PP, Sacchetti R, Stampi S, Zanetti F (2005). *Legionella* waterline colonization: detection of *Legionella* species in domestic, hotel and hospital hot water systems. J Appl Microbiol.

[10] Nakadate T, Yamaguchi K, Inoue H (1999). An outbreak of Legionnaires’ disease associated with a Japanese spa. Nihon kokyuki Gakkai Zasshi.

[11] Okada M, Kawano K, Kura F, Amemura-Maegawa J, Watanabe H, Yagita K, Endo T, Suzuki S (2005). The largest outbreak of Legionellosis in Japan associated with spa baths: epidemic curve and environmental investigation. J Jpn Assoc Infect Dis.

[12] Solhang A, Bergh K (2006). Identification and differentiation of *Legionella pneumophila* and *Legionella* spp. with real-time PCR targeting the 16S rRNA gene and species identification by *mip* sequencing. Appl Environ Microbiol.

[13] Tanaka K, Yamaguchi N, Baba T, Amano N, Nasu M (2011). Rapid enumeration of low numbers of moulds in tea based drinks using an automated system. Int J Food Microbiol.

[14] Wang X, Yamaguchi N, Someya T, Nasu M (2007). Rapid and automated enumeration of viable bacteria in compost using a micro-colony auto counting system. J. Microbiol. Methods.

[15] Wery N, Bru-Adan V, Minervini C, Delgénes JP, Garrelly L, Godon JJ (2008). Dynamics of *Legionella* spp. and bacterial populations during the proliferation of *L. pneumophila* in a cooling tower facility. Appl Environ Microbiol.

[16] Wu S, Ueno D, Inoue K, Someya T (2009). Direct viable count combined with fluorescence in situ hybridization (DVC-FISH) for specific enumeration of viable *Escherichia coli* in cow manure. Microbes Environ.

